# Optimization of a chest computed tomography protocol for detecting pure ground glass opacity nodules: A feasibility study with a computer-assisted detection system and a lung cancer screening phantom

**DOI:** 10.1371/journal.pone.0232688

**Published:** 2020-05-22

**Authors:** Seongmin Kang, Tae Hoon Kim, Jae Min Shin, Kyunghwa Han, Ji Young Kim, Baeggi Min, Chul Hwan Park

**Affiliations:** 1 Department of Radiology and the Research Institute of Radiological Science, Gangnam Severance Hospital, Yonsei University College of Medicine, Seoul, Republic of Korea; 2 Department of Radiology and the Research Institute of Radiological Science, Severance Hospital, Yonsei University College of Medicine, Seoul, Republic of Korea; 3 GE healthcare, Seongnam-si, Korea; University of California Berkeley, UNITED STATES

## Abstract

**Objective:**

This study aimed to optimize computed tomography (CT) parameters for detecting ground glass opacity nodules (GGNs) using a computer-assisted detection (CAD) system and a lung cancer screening phantom.

**Methods:**

A lung cancer screening phantom containing 15 artificial GGNs (−630 Hounsfield unit [HU], 2–10 mm) in the left lung was examined with a CT scanner. Three tube voltages of 80, 100, and 120 kVp were used in combination with five tube currents of 25, 50, 100, 200, and 400 mA; additionally, three slice thicknesses of 0.625, 1.25, and 2.5 mm and four reconstruction algorithms of adaptive statistical iterative reconstruction (ASIR-V) of 30, 60, and 90% were used. For each protocol, accuracy of the CAD system was evaluated for nine target GGNs of 6, 8, or 10 mm in size. The cut-off size was set to 5 mm to minimize false positives.

**Results:**

Among the 180 combinations of tube voltage, tube current, slice thickness, and reconstruction algorithms, combination of 80 kVp, 200 mA, and 1.25-mm slice thickness with an ASIR-V of 90% had the best performance in the detection of GGNs with six true positives and no false positives. Other combinations had fewer than five true positives. In particular, any combinations with a 0.625-mm slice thickness had 0 true positive and at least one false positive result.

**Conclusion:**

Low-voltage chest CT with a thin slice thickness and a high iterative reconstruction algorithm improve the detection rate of GGNs with a CAD system in a phantom model, and may have potential in lung cancer screening.

## Introduction

The term ‘subsolid nodules (SSNs)’ include both pure ground-glass nodules (GGNs) and part-solid nodules [1]. A GGN is a focal nodular lesion with increased lung attenuation through which normal lung architecture can be observed. A part-solid nodule includes both ground-glass and solid components [1,2]. In lung cancer screening, up to 19% of the pulmonary nodules detected on baseline studies are SSNs [3,4]. Although 37–70% of the SSNs are transient according to previous studies [3,5,6], persistent SSNs identified on computed tomography (CT) have a higher possibility of malignancy than solid nodules. Persistent SSNs detected on screening have a malignancy rate of up to 34%; the previously reported malignancy rate of GGNs and part-solid nodules was 18 and 63%, respectively [4,7]. Therefore, early detection of SSNs and continuous follow-up could be important factors in lung cancer prognosis. However, visualization of SSNs can be easily missed on CT screens, and according to Li et al., 91% of missed lung cancer lesions on CT were SSNs [8,9].

A computer-assisted detection (CAD) system detects a specific lesion in an image using a computer algorithm [10]. On chest CT, CAD systems can act as second readers in the identification of missed nodules and show good sensitivity for detecting small lung cancers [11–14]. Moreover, CAD systems can reduce the time required to detect lung nodules, and therefore, could be useful for early lung cancer screening on chest CT scans. However, CAD systems have limited value in the automated detection of SSNs. In particular, GGNs are not well detected by CAD systems because of small differences in attenuation compared to the lung parenchyma [15].

Therefore, optimizing the CT parameters is an important factor in increasing the accuracy of the CAD system for identifying SSNs, particularly GGNs. However, limited studies have been conducted on this topic. Therefore, in this study, we conducted a preliminary study to optimize the CT parameters to detect GGNs using a CAD system and a lung cancer screening phantom.

## Materials and methods

### Phantom and target lesion

**Fig 1:**
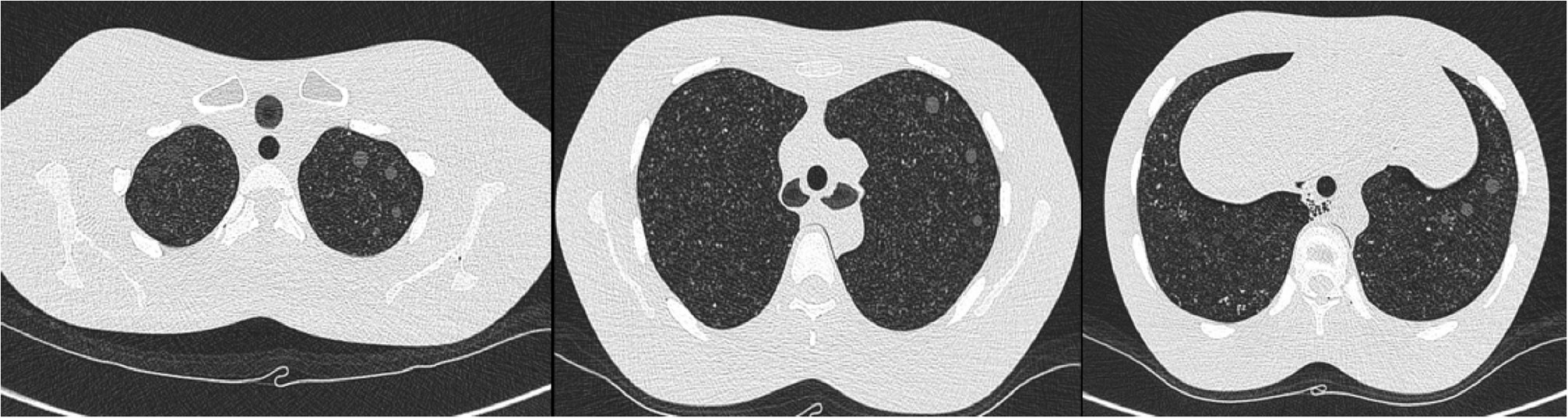
Nine target ground glass nodules (GGNs) in the left lung of lung screening phantom. A lung screening computed tomography phantom was used. Attenuation of the lung parenchyma was **−**900 Hounsfield unit. Of the 15 artificial GGNs in the left lung, nine lesions (6-, 8-, or 10-mm nodules at the lung apex, tracheal bifurcation, and base of the lung) were used as targets for the computer-assisted detection system.

A lung screening CT (LSCT) phantom (LSCT-001 type phantom, Kyoto Kagaku Co., Ltd., Kyoto, Japan) was used for optimization of the CT protocol to identify GGNs. Several previous phantom studies used this phantom for the detection of GGNs [16–19]. The phantom represents the chest of an adult male and is made of materials equivalent to the consistency of human tissues. Attenuation of the lung parenchyma is designed to be −900 Hounsfield unit (HU). There are 30 artificial GGNs (15 in right lung: −800 HU, 4–12 mm in 2-mm increments; 15 in left lung: −630 HU, 2–10 mm in 2-mm increments) distributed at the lung apex, tracheal bifurcation, and base of the lung (five nodules in each location) in both lung fields. Among them, nine lesions in the left lung (−630 HU), 6, 8, or 10 mm in size, were defined as the target lesions (Fig 1).

### CT protocol

All CT scans were performed with a 256-row multi-detector CT scanner (Revolution CT, General Electric, Boston, MA, US). Chest CT was performed using a helical technique and a mediastinal window setting with the following exposure parameters: Three tube voltages of 80, 100, and 120 kVp in combination with five tube currents of 25, 50, 100, 200, and 400 mA. The data were reconstructed with three slice thicknesses of 0.625, 1.25, and 2.5 mm; four different reconstruction algorithms of adaptive statistical iterative reconstruction (ASIR-V) of 30, 60, and 90% on the scanner workstation. Each of the variables was determined by dividing the parameters in the clinically available range into 3~5 sections. All CT images were transferred to the picture archiving and communication system (Centricity 2.0; GE Medical Systems) and CAD system for analysis.

### CT image analysis with computer-assisted detection system

**Fig 2:**
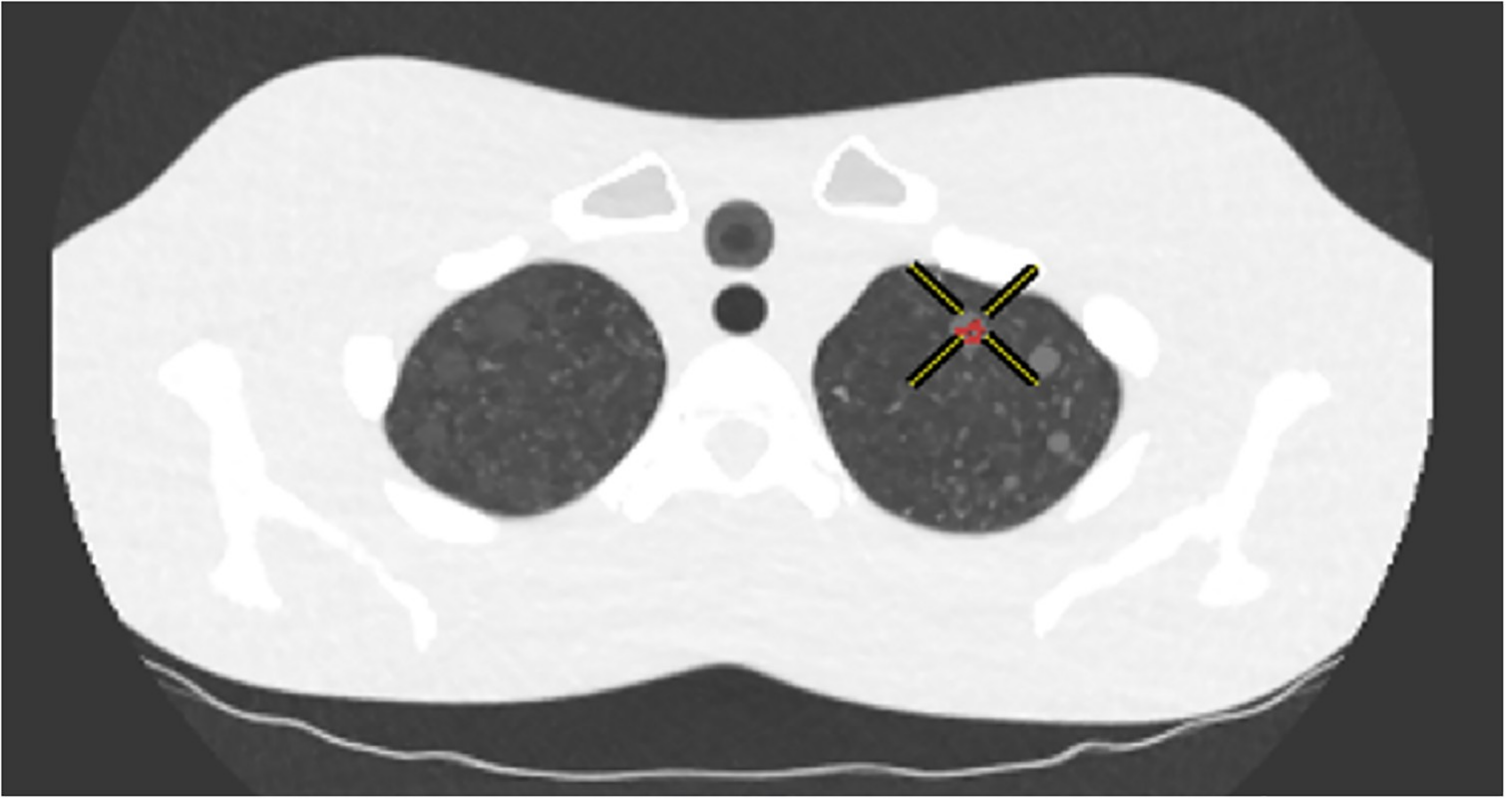
Example of true positive detection.

**Fig 3:**
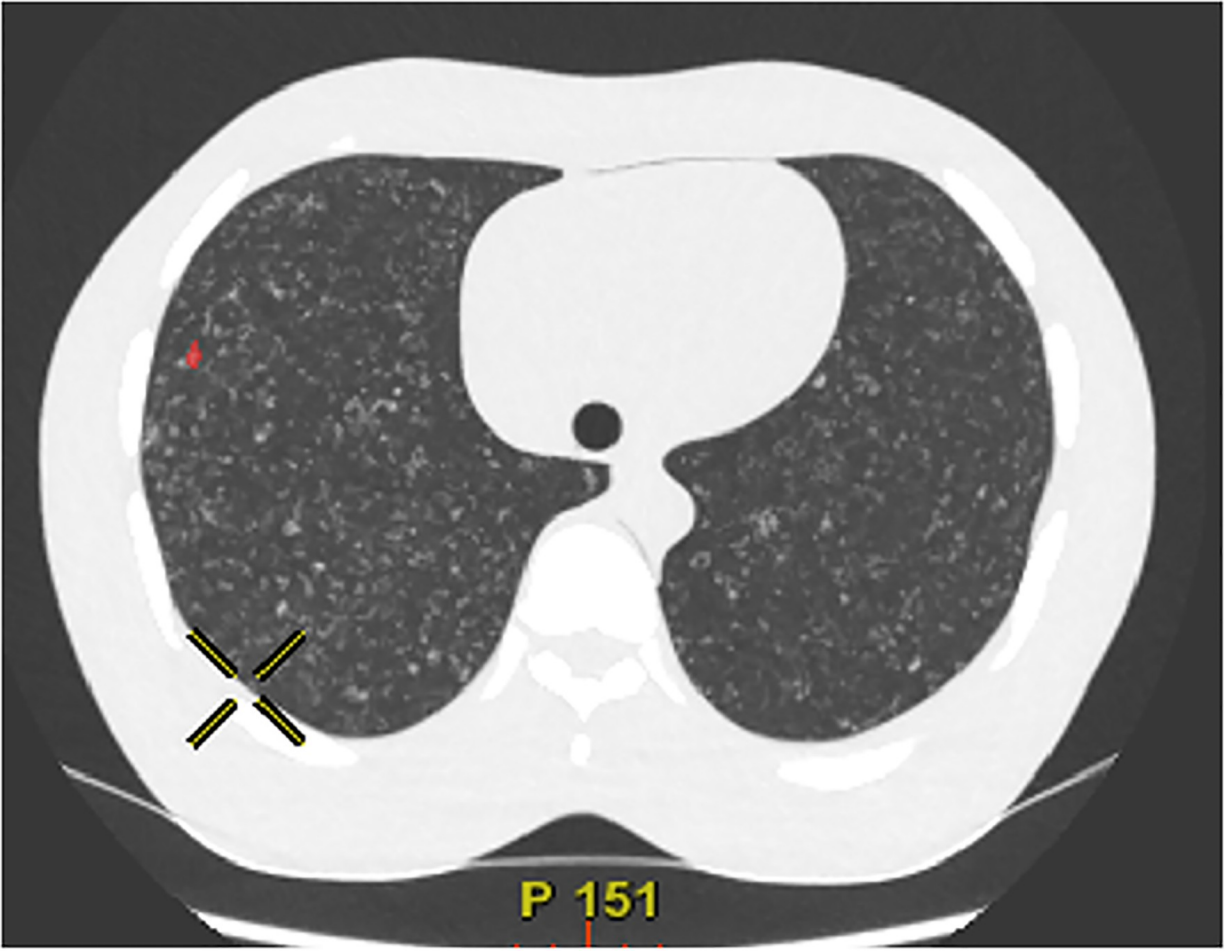
Example of false positive detection.

Two radiologists (THK and CHP, with 22 and 10 years of experience in chest radiology, respectively) analysed the CT scans. The CAD system used in this study was Lung VCAR from GE healthcare. For each protocol, the accuracy of the CAD system was evaluated for the nine target GGNs that were 6, 8, or 10 mm in size and present in the left lung (Figs 2 and 3). The cut-off size of the CAD system was set to 5 mm to minimize false positives (FPs).

### Statistical analyses

Three factors were used to evaluate the detection accuracy for each parameter. First, the true-positive rate (TPR) was defined as the percentage of the total number of nodules identified using each parameter divided by the total number of nodules in each parameter. Second, the number of FP per examination result was defined as the sum of identified false positive nodules per examination of each parameter. Lastly, we determined the optimal combination of parameters for the detection of nodules. For statistical analysis, logistic regression using the generalized estimating equation method, including the main effects for each CT parameter, was used (95% confidence interval for the TPR and comparative p-value). All analyses were performed by SAS (version 9.4, SAS Institute Inc.) software.

## Results

### Tube voltage

There were 60 combinations in each tube voltage, and the number of true positive nodules and FP nodules found at each tube voltage were obtained (Table 1). At tube voltages of 80, 100, and 120 kVp, the TPR was 14.81%, 9.81%, and 6.67%, respectively, with the highest TPR was obtained at 80 kVp. The number of FPs per examination was 0.36, 0.67, and 0.77at 80, 100, and 120 kVp, respectively, with the lowest number of FPs were observed at 80 kVp. As the tube voltage increased, the number of FP per examination increased significantly, but the TPR decreased significantly. Pairwise comparison of the TPR and FP revealed significant differences (p = 0.001) with the exception of those obtained at 100- and 120-kVp tube voltages.

**Table 1:** Results of computer-assisted detection system according to the tube voltages.

Tube voltage(kVp)	True positive nodules (n)	True positive rate (%, 95% CI)	False positive nodules (n)	No. of false-positives per examination (n)
80	80	14.81 (11.3, 19.19)	22	0.36
100	53	9.81 (7.34, 13.01)	40	0.67
120	36	6.67 (4.66, 9.46)	46	0.77
**p-value for tube voltages**		<.0001		<.0001
***Pairwise comparison (p-values)***				
80 vs. 100		<.0001		<.0001
80 vs. 120		<.0001		<.0001
100 vs. 120		0.0148		0.2334

CI, confidence interval

### Tube current

There were 36 combinations for each tube current, and the number of true positive nodules and FP nodules found at each tube current were obtained (Table 2). When the tube current was 25, 50, 100, 200, and 400 mA, the TPR was 7.72%, 7.72%, 11.73%, 11.11%, and 13.89%, respectively. As the tube current increased, the TPR tended to increase significantly. Pairwise comparison of the TPR for each tube current indicated that the difference was significant in the comparisons of 25 mA vs. 100 mA, 25 mA vs. 400 mA, 50 mA vs. 100 mA, 50 mA vs. 200, 50 mA vs. 400 mA, and 200 mA vs. 400 mA. However, the number of FP per phantom did not significantly differ between each tube currents (number of FPs per examination was 0.67, 0.67, 0.56, and 0.56 with tube currents of 25, 50, 100, 200, and 400 mA).

**Table 2:** Results of computer-assisted detection system according to the tube currents.

tube current (mA)	True positive nodules (n)	True positive rate (%, 95% CI*)	False positive nodules (n)	No. of false-positives per examination (n)
25	25	7.72 (4.93, 11.88)	24	0.67
50	25	7.72 (4.93, 11.88)	24	0.67
100	38	11.73 (7.96, 16.95)	20	0.56
200	36	11.11 (7.27, 16.62)	20	0.56
400	45	13.89 (10.05, 18.89)	20	0.56
**p-value for tube currents**		0.0004		0.5264
***Pairwise comparison* (p-values)**				
25 vs. 50		>.9999		NA
25 vs. 100		0.0224		NA
25 vs. 200		0.1212		NA
25 vs. 400		0.0070		NA
50 vs. 100		0.0005		NA
50 vs. 200		0.0248		NA
50 vs. 400		0.0009		NA
100 vs. 200		0.6393		NA
100 vs. 400		0.1615		NA
200 vs. 400		0.0437		NA

NA, Not applicable; CI, confidence interval

### Slice thickness

There were 60 combinations for each slice thickness. The TPR and number of FP were obtained by counting the number of true positive and FP nodules in each slice thickness (Table 3). When the slice thickness was 0.625, 1.25, and 2.5 mm, the TPR was 0.37%, 14.44%, and 16.48%, respectively. When the slice thickness was 0.625 mm, the number of true positive nodules was two, indicating that the CAD system did not identify most GGNs. When comparing the slice thicknesses of 1.25 mm and 2.5 mm, there was no significant difference. The number of FP per examination in each slice thickness was 0.97, 0.7, and 0.13 for slice thicknesses of 0.625, 1.25, and 2.5 mm, respectively. The number of FP per examination was significantly lower at a slice thickness of 2.5 mm, and there was no significant difference between slice thicknesses of 0.625 mm and 1.25 mm.

**Table 3:** Results of computer-assisted detection system according to the slice thickness.

Slice thickness (mm)	True positive nodules (n)	True positive rate (%, 95% CI)	False positive nodules (n)	No. of false-positives per examination (n)
0.625	2	0.37 (0.09, 1.44)	58	0.97
1.25	78	14.44 (10.82, 19.02)	42	0.7
2.5	89	16.48 (14.5, 18.67)	8	0.13
**p-value for slice thickness**		<.0001		<.0001
***Pairwise comparison***				
0.625 vs. 1.25		<.0001		0.010
0.625 vs. 2.5		<.0001		<.0001
1.25 vs. 2.5		0.2468		<.0001

CI, confidence interval

### Reconstruction methods

There were 45 combinations for each reconstruction method (Table 4). When the ASIR-V was 0, 30, 60, and 90%, the TPR was 5.19%, 7.41%, 11.85%, and 17.28%, respectively. As the ASIR-V value increased, the TPR significantly increased. In cases of FP nodules, there was no significant difference in the ASIR-V between the 33, 24, 22, and 29 FP nodules identified at 0, 30, 60, and 90%, respectively.

**Table 4:** Results of computer-assisted detection system according to the reconstruction methods.

ASIR_V	True positive nodules (n)	True positive rate (%, 95% CI)	False positive nodules (n)	No. of false-positives per examination (n)
0 (filtered back projection)	21	5.19 (3.51, 7.6)	33	0.73
30	30	7.41 (5.22, 10.41)	24	0.53
60	48	11.85 (8.76, 15.84)	22	0.49
90	70	17.28 (12.83, 22.88)	29	0.64
**p-value for ASIR_V**		<.0001		0.0198
***Pairwise comparison***				
0 vs. 30		0.0016		0.0040
0 vs. 60		<.0001		0.0067
0 vs. 90		<.0001		0.3584
30 vs. 60		<.0001		0.4837
30 vs. 90		<.0001		0.2266
60 vs. 90		<.0001		0.0883

CI, confidence interval

### Best combination

Among the 180 combinations of tube voltage, tube current, slice thickness, and reconstruction algorithms, 80 kVp, 200 mA, and 1.25 mm slice thickness with an ASIR-V of 90% had the best performance in the detection of GGNs with six true positives and no FPs. For 80 kVp, 400 mA, and 1.25-mm slice thickness with an ASIR-V of 90%, there were five true positives and one FP; for 100 kVp, 100 mA, and 1.25-mm slice thickness with an ASIR-V of 90%, there were four true positives and one FP; 100 kVp, 50 mA, and 1.25-mm slice thickness with an ASIR-V of 90% resulted in four true positives and one FP; and for 120 kVp, 25 mA, and 1.25-mm slice thickness with an ASIR-V of 90%, there were four true positives and three FPs (Table 5). Other combinations had fewer than three true positives. In particular, any combinations with a slice thickness of 0.625 mm had 0 true positive and at least one FP result [S1 File].

**Table 5:** Five combinations of tube voltage, tube current, slice thickness, and reconstruction algorithms.

Tube voltage (kVp)	Tube current (mAs)	Slice thickness (mm)	ASIR_V	True-positive nodules (n)	False-positive nodules (n)
80	200	1.25	90	6	0
80	400	1.25	90	5	1
100	100	1.25	90	4	1
100	50	1.25	90	4	1
120	25	1.25	90	4	3

## Discussion

In the present study, chest CT at a low voltage with a thin slice thickness and a high iterative reconstruction algorithm improved the detection rate of a CAD system for small GGNs in a phantom model. However, overly thin slices hampered the CAD system.

### Tube voltage

In our study, as the tube voltage increased, the TPR decreased and the number of FPs increased significantly. Therefore, according to our results, the detection rate of GGNs using the CAD algorithm decreases as the tube voltage increases. Considering the photoelectric effect, the contrast between nodules and the lung parenchyma increases as the tube voltage decreases. Because the probability of the photoelectric effect is proportional to Z^3^ (atomic number), contrast increases with the atomic number of the nodule [20]. However, the artificial GGNs in our study comprised low atomic number materials; therefore, photoelectric effect would have little impact on the contrast between GGNs and the parenchyma [21,22].

### Tube current

According to our result, as the tube current increased, the TPR tended to increase significantly but the number of FPs did not significantly differ. The image noise decreases as the tube current increases, and when the tube voltage, slice thickness, and reconstruction method are fixed, the contrast-to-noise ratio (CNR) tends to increase and the noise tends to decrease significantly with increasing tube current [23].

### Slice thickness

As the slice thickness increased, both the TPR and number of FPs increased, indicating that the detection rate increased with increasing slice thickness (Table 3). The difference in detection rate according to slice thickness can be explained by spatial resolution and noise. As the slice thickness increases, the spatial resolution decreases and the sharpness between the pulmonary nodule and the parenchyma decreases. However, as the slice thickness increases, the noise tends to decrease [23]. In our study, the detection rate tended to increase with increasing slice thickness, number of false positive nodules per exam also decrease with increase slice thickness Therefore, noise may be more influential than spatial resolution for the detection of GGNs by the present CAD algorithm.

### Reconstruction methods

As the ASIR-V value increased, the TPR was significantly increased and there was no significant difference in the number of FPs. In the study by Yanagawa et al. [24], the sensitivity of a CAD system for identifying pulmonary nodules was 54, 61, and 71 for an ASIR-V of 0, 50, and 100% in patients’ clinical routine-dose chest CT Using an iterative reconstruction algorithm could not only increase the signal-to-noise ratio, but also enhance the sharpness of the margins between nodules and the lung parenchyma, resulting in increased sensitivity of the CAD program. According to Daiele et al. [25], the CNR was significantly higher on abdomen CT with an ASIR algorithm than on abdomen CT with standard filtered back projection (FBP) reconstruction in real patients. In addition, Solomon et al. [23]. reported that the noise on a chest CT of a phantom was decreased when applying iterative reconstruction compared to chest CT with standard FBP reconstruction.

### Optimal combination and future directions

Research on early lung cancer screening by detecting SSNs using the CAD algorithm has been limited. GGNs, in particular, are more difficult to detect using the CAD algorithm because of the small difference in attenuation with regard to the lung parenchyma. Benzakoun et al.[15] evaluated the performance of a CAD system in the automated detection and measurement of subsolid nodules. According to this study, a CAD system has limited value for the automated detection of GGNs. The study compared the detection rate for part-solid GGNs and pure GGNs, which was 36/50 (72%) and 14/50 (28%), respectively, at 3-mm slice thickness (p<0.0001). Thus, the detection rate for pure GGNs is not high according to Benzakoun et al. [15] and the detection rate for pure GGN on lung phantom using the CAD algorithm is not high according to our study (for example, the TPR of the combinations using an ASIR-V of 90% was 17.28%). However, the detection rate of the optimal combination (tube voltage of 80 kVp, tube current of 200 mA, slice thickness of 1.25 mm, and ASIR-V of 90%) was 67% (six out of nine GGNs). Therefore, when the results of this optimal combination are applied to GGN detection in actual patients, the results then might be better than those of previous studies. Thus, further studies applying our findings to the detection of GGNs in real patients are warranted.

### Limitations

There are some limitations to our study. First, our study aimed to detect artificial GGNs in a phantom. Therefore, it is uncertain whether these results could be applied to the detection of GGNs in actual patients or in solid nodules. In daily practice, there are both solid and subsolid nodules, which might have different optimal CT parameters. Some parameters, such as slice thickness and iterative reconstruction, could be changed after image acquisition. However, some parameters, such as tube voltage or tube current, should be selected prior to CT scanning. However, we focused on optimizing the CT parameters for GGNs because there have been several studies that have shown that the CAD detection rate is adequate for detecting solid nodules. Further studies are required that evaluate the optimized CT parameters for solid and subsolid nodules simultaneously. Second, in our study, GGNs <5 mm were not detected because of the characteristics of the lung VCAR CAD program, which can only detect nodules having a size of more than twice the slice thickness. However, in real practice, both solitary GGNs and part-solid nodules <6 mm in size are not clinically significant [26]; therefore, CAD can be used to detect clinically significant SSNs that are >5 mm. Third, we used only one type of CAD system to detect GGNs. Lastly, the tendency of the TPR to decrease and number of FPs to increase as the tube voltage increases is the opposite to what we expected and cannot be explained by existing results. However, if this tendency can be applied equally to GGN detection using the CAD system in real patients, the CAD system can be used to detect GGNs in low dose CT screening.

## Conclusion

Low-voltage chest CT with a thin slice thickness and a high iterative reconstruction algorithm might improve the detection rate of a CAD system for small GGNs in a phantom model, and may have potential in lung cancer screening.

## Supporting information

S1 File Attached file contains data for true positive and false positive nodules detected by the CAD system at each combination of CT parameters. (XLSX)

## Abbreviations

ASIR-V: adaptive statistical iterative reconstruction
CAD: computer-assisted detection
CNR: contrast-to-noise ratio
FP: false positive
FBP: filtered back projection
GGN: ground-glass nodule
LSCT: lung screening CT
SSN: Subsolid nodule
TPR: true-positive rate
